# Improving performance of deep learning models using 3.5D U-Net via majority voting for tooth segmentation on cone beam computed tomography

**DOI:** 10.1038/s41598-022-23901-7

**Published:** 2022-11-17

**Authors:** Kang Hsu, Da-Yo Yuh, Shao-Chieh Lin, Pin-Sian Lyu, Guan-Xin Pan, Yi-Chun Zhuang, Chia-Ching Chang, Hsu-Hsia Peng, Tung-Yang Lee, Cheng-Hsuan Juan, Cheng-En Juan, Yi-Jui Liu, Chun-Jung Juan

**Affiliations:** 1 Department of Periodontology, School of Dentistry, Tri-Service General Hospital, National Defense Medical Center, Taipei, Taiwan, ROC; 2 School of Dentistry and Graduate Institute of Dental Science, National Defense Medical Center, Taipei, Taiwan, ROC; 3 Department of Medical Imaging, Xinglong Rd, China Medical University Hsinchu Hospital, 199, Sec. 1, Zhubei, 302 Hsinchu Taiwan, ROC; 4 Ph.D. Program in Electrical and Communication Engineering, Feng Chia University, Taichung, Taiwan, ROC; 5 Department of Automatic Control Engineering, Feng Chia University, No. 100 Wenhwa Rd., Seatwen, 40724 Taichung Taiwan, ROC; 6 Master’s Program of Biomedical Informatics and Biomedical Engineering, Feng Chia University, Taichung, Taiwan, ROC; 7 Department of Management Science, National Yang Ming Chiao Tung University, Taipei, Taiwan, ROC; 8 Department of Biomedical Engineering and Environmental Sciences, National Tsing Hua University, Hsinchu, Taiwan, ROC; 9 Cheng Ching Hospital, Taichung, Taiwan, ROC; 10 Department of Radiology, School of Medicine, College of Medicine, China Medical University, Taichung, Taiwan, ROC; 11 Department of Medical Imaging, China Medical University Hospital, Taichung, Taiwan, ROC; 12 Department of Biomedical Engineering, National Defense Medical Center, Taipei, Taiwan, ROC; 13 Department of Computer Science and Information Engineering, National Taiwan University, Taipei, Taiwan, ROC

**Keywords:** Dentine, Enamel, Biomedical engineering

## Abstract

Deep learning allows automatic segmentation of teeth on cone beam computed tomography (CBCT). However, the segmentation performance of deep learning varies among different training strategies. Our aim was to propose a 3.5D U-Net to improve the performance of the U-Net in segmenting teeth on CBCT. This study retrospectively enrolled 24 patients who received CBCT. Five U-Nets, including 2Da U-Net, 2Dc U-Net, 2Ds U-Net, 2.5Da U-Net, 3D U-Net, were trained to segment the teeth. Four additional U-Nets, including 2.5Dv U-Net, 3.5Dv5 U-Net, 3.5Dv4 U-Net, and 3.5Dv3 U-Net, were obtained using majority voting. Mathematical morphology operations including erosion and dilation (E&D) were applied to remove diminutive noise speckles. Segmentation performance was evaluated by fourfold cross validation using Dice similarity coefficient (DSC), accuracy, sensitivity, specificity, positive predictive value (PPV), negative predictive value (NPV). Kruskal–Wallis test with post hoc analysis using Bonferroni correction was used for group comparison. *P* < 0.05 was considered statistically significant. Performance of U-Nets significantly varies among different training strategies for teeth segmentation on CBCT (*P* < 0.05). The 3.5Dv5 U-Net and 2.5Dv U-Net showed DSC and PPV significantly higher than any of five originally trained U-Nets (all *P* < 0.05). E&D significantly improved the DSC, accuracy, specificity, and PPV (all *P* < 0.005). The 3.5Dv5 U-Net achieved highest DSC and accuracy among all U-Nets. The segmentation performance of the U-Net can be improved by majority voting and E&D. Overall speaking, the 3.5Dv5 U-Net achieved the best segmentation performance among all U-Nets.

## Introduction

Cone beam computed tomography (CBCT) has been widely applied to orthodontics, periodontics, endodontics, stomatology, dental implant surgery, maxillofacial surgery, and forensic odontology^1,2^. It is superior to panoramic radiography and periapical radiography by providing 3D information rather than 2D information and has advantages over conventional CT including, but not limited to, lower radiation doses and lower costs.

Rapid, accurate, and robust segmentation of human teeth on CBCT is an important foundation of clinical practice in dentistry. It allows clear visualization of teeth on the one hand, and, is helpful for qualitative evaluation and quantitative analysis of dental diseases such as caries^3,4^, impacted tooth^5^, acute pulpitis^6^, apical periodontitis^7^, root fracture and periodontal lesion^4^. Manual segmentation by experts is usually considered as gold standard. However, it is laborious and time-consuming with the segmentation performance varying among different experts^8^. Semiautomatic segmentation facilitates the process of segmentation and is less laborious and less time-consuming with comparable segmentation performance with manual segmentation^9,10^. Automatic segmentation outperforms manual and semiautomatic segmentation by providing rapidest and most efficient segmentation of teeth^11^. However, automatic segmentation has been shown inferior to manual segmentation and semiautomatic segmentation in calculating tooth volume using water displacement method as gold standard^9^. In addition, automatic segmentation of teeth on CBCT remains challenging because of the more severe artifacts such as beam hardening artifacts^12,13^, unsharpness^12–14^, ring-like artifacts^13,14^, partial volume averaging^13^, undersampling^13^, cone-beam effect^13,14^, noises^15^, aliasing artifacts, and poorer soft-tissue contrast as compared to conventional CT^16^.

Deep learning is a subset of machine learning. Encouraged by the human neural structures, deep learn learns to think as the human brain by implementing multi-layer artificial neural networks. Supervised learning is the most common form of deep learning although the learning can also be semi-supervised or unsupervised. By feeding labeled data, including but not limited to images, into the complex and non-linear neural networks, deep learning works mimicking the human neural networks and gives results that enable us to detect, classify, and segment objects in interest^17^. Recently deep learning has a lot of attention because it can perform as good as human and even better in specific tasks.

First proposed in 2015 by Ronneberger et al.^18^, U-Net has been widely applied for medical imaging segmentation because it provides context information using fewer time and smaller data to train^19^. The U-Net contains a contraction path and an expansion path to encode the data using convolution and decode the data using up-convolution, respectively. It also concatenates the encoder and decoder by copying and cropping the input image to match the size of feature maps between the encoder and decoder layer by layer so that the net can not only classify but also localize the object for segmentation.

Several U-Nets including 2D U-Net^20,21^, 2.5D U-Net^22^, and 3D U-Net^23^ have been proposed for CBCT segmentation. A variant of 2.5D U-Net using majority voting of 2D U-Nets trained by 3 orthogonal imaging planes has been shown to outperform any single U-Net for maxillary and mandibular bony structure segmentation on CBCT^24^. To the best of our knowledge, CT using a 3.5D U-Net integrating 2D U-Nets, 2.5D U-Net, and 3D U-Net has never been documented yet.

We hypothesized that the segmentation performance of a 3.5D U-Net might be improved using majority voting by reducing the false positive results occurring in 2D U-Net, 2.5D U-Net and 3D U-Net. In this study, we intentionally applied 6 previously introduced U-Nets including three orthogonal 2D U-Nets, two 2.5D U-Nets, plus a 3D U-Net and added three newly proposed 3.5D U-Nets by integrating 2D U-Nets, 2.5D U-Nets and 3D U-Net using the majority voting method for segmentation of teeth on CBCT. The proposed 3.5D U-Nets were compared to the previous U-Nets using slice-by-slice calculation of Dice similarity coefficient (DSC) and other diagnostic metrics including accuracy (Ac), sensitivity (Sn), specificity (Sp), positive predictive value (PPV), and negative predictive value (NPV) to verify our hypothesis.

## Materials and methods

This study was approved by the Institutional Review Board of China Medical University with written informed consent waived for this retrospective study. All methods were performed in accordance with the relevant guidelines and regulations.

### Patient cohort and CBCT parameters

**Figure 1:**
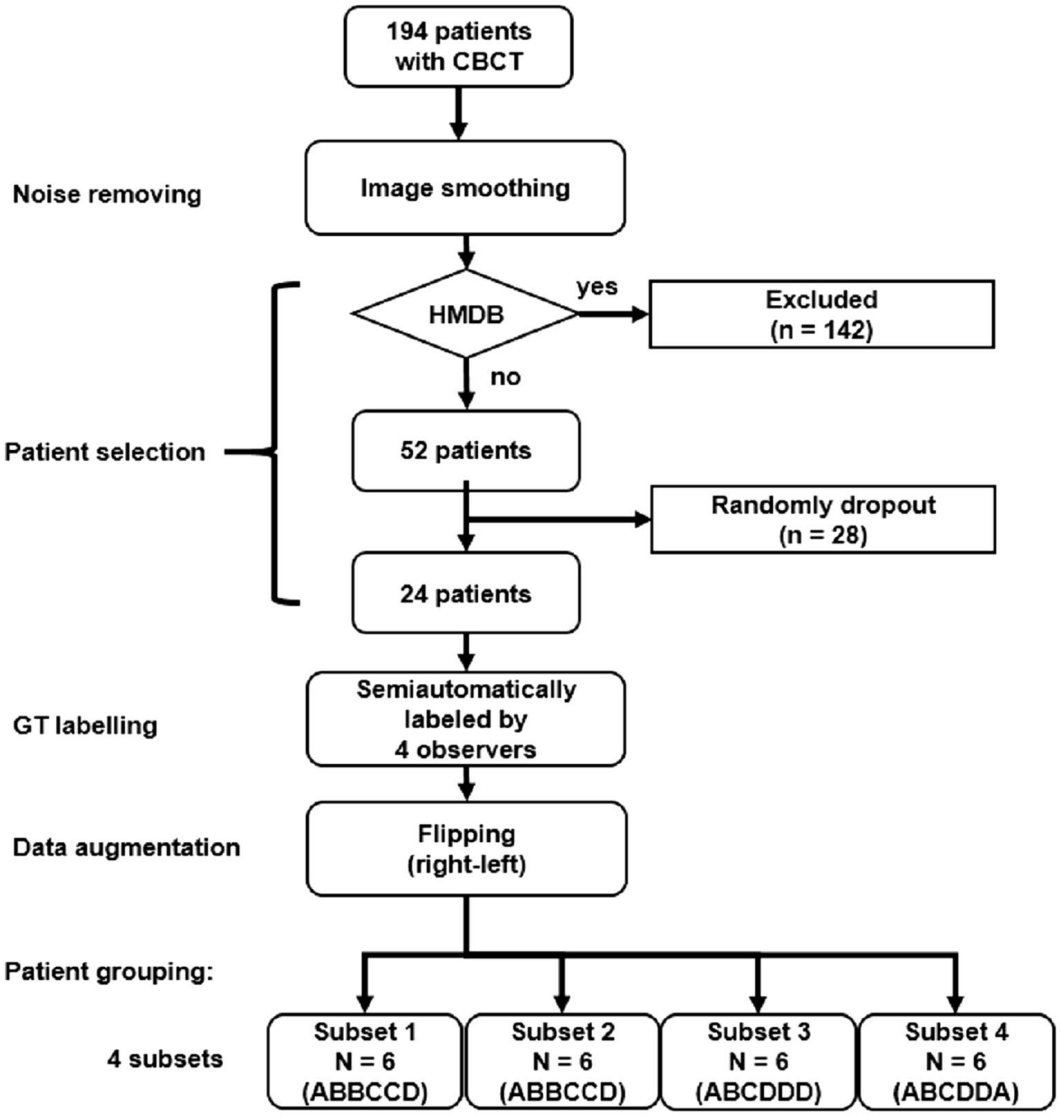
Flowchart describing noise removing, patient selection, GT labeling, data augmentation and patient grouping of this study. CBCT denotes cone beam computed tomography, GT denoted ground truth, and HMDB denotes heavy metallic dental burden. ABCD in subsets denotes observer A, B, C, D, respectively.

Figure 1 demonstrates the processes from noise removing, patient selection, GT labeling, data augmentation and patient grouping in our study. A total of 194 patients who received CBCT study from January to June 2020 were initially collected. All patients were scanned using an Auge Solio CBCT scanner (Asahi Roentgen Ind., Kyoto, Japan) that is widely used in dentistry and maxillofacial surgery. All scans were performed using a tube voltage of 85 kVp, a tube current of 6 mA, and an isotropic voxel size of 0.19 mm. The imaging protocol covered from the inferior orbital rim to the inferior end of the mandible.

In order to minimize the potential influence of metal-related artifacts on the segmentation task, one of our exclusion criteria was patients with heavy metallic dental burden (MDB) including metallic dental implants, braces and crowns. CBCTs with heavy MDB due to metallic dental devices were automatically identified according to the following steps and excluded. First, two thresholds were empirically set with the first threshold (TH1) of 3070 HU and the second threshold (TH2) of 2500 HU, representing the density of metallic materials and the density of enamel, respectively. Second, MDB ratio (MDBR) was defined via dividing TH1 by TH2. Third, a third threshold (TH3) was set with the MDBR = 0.4. Fourth, heavy MDB was defined by MDBR > TH3. Fifth, patients with heavy MBD were excluded. A total of 24 patients were randomly selected from the rest of patients for segmentation of teeth in this study to prevent huge loading of manpower in defining the ground truth (GT). Patients were classified into 4 subsets, in which each subset containing same number of patients (N = 6) with the GT defined by different observers.

### Imaging preprocessing

In order to remove high frequency noise in CBCT, a 3D Gaussian filter with standard deviation of 1 was applied first. All teeth were slice-by-slice contoured semiautomatically on CBCT by four different observers including one dentist (K.H. with 6-year experience in medical imaging research) and three researchers majoring in medical imaging analysis (P.S.L., G.X.P. and Y.C.Z. with one more year of experience in medical imaging analysis). The semiautomatic method is modified from that used in our previous study using thresholding method^25^. First, the CBCT images were loaded and displayed. Second, a polygonal region-of-interest (ROI) encompassing teeth was drawn. Third, a threshold was initially applied and then adjusted to fit the contour of teeth. Four, holes within the contour of teeth were filled. Finally, all images with teeth successfully contoured were save as GT. All GTs were verified by a neuroradiologist (C.J.J. with more than 20 years of experience in medical imaging analysis).

Data augmentation with an augmentation factor of 2 was achieved by flipping all images along the horizontal direction. For fair comparison among the original U-Nets, no additional data augmentation was performed for either 2.5D U-Net or 3D U-Net.

### Deep learning models (DLMs)

U-Net was employed for semantic segmentation of teeth in this study^18^. The U-Net architecture consists of a decoding path and an encoding path symmetrically. The decoding path contains two convolution blocks in each layer with each convolution block followed by a rectified linear unit (Relu) to obtain lower-dimensional representation and then down-sampled by a max pooling operation. In the encoding path, the representation is concatenated with the corresponding features maps obtained in the encoding path, followed by two convolution blocks, and then up-sampled by nearest convolution operation. The final output layer of the U-Net was connected to a dual-class softmax classifier, i.e., teeth and non-teeth.

**Figure 2:**
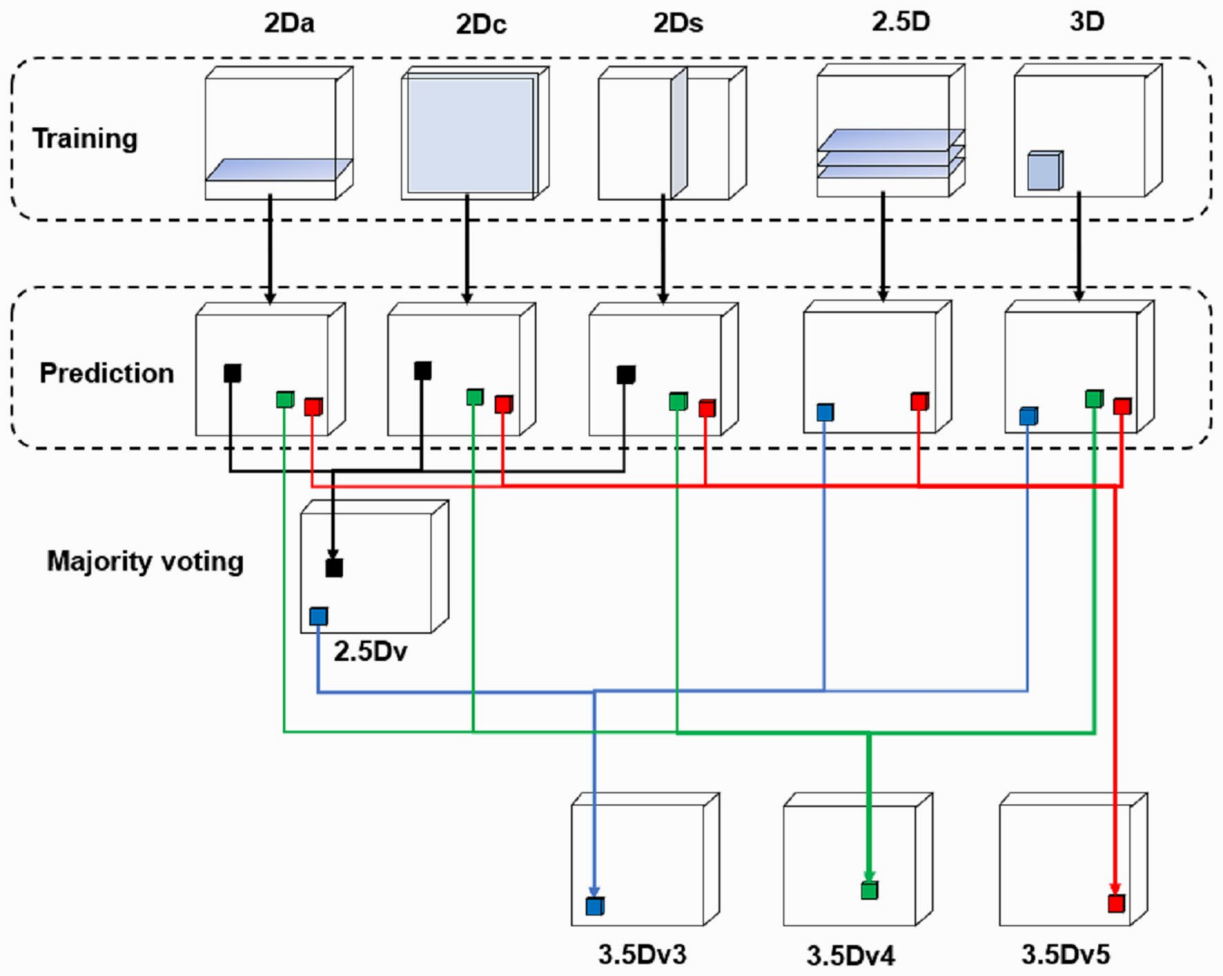
Schematics of the 2.5D U-Net and our proposed 3.5D U-Nets using majority voting. The 2.5D U-Net combines the predictions of deep learning models trained by 2Da U-Net, 2Dc U-Net and 2Ds U-Net. The 3.5Dv3 U-Net combines the predictions of deep learning models trained by 2.5Dv U-Net, 2.5D U-Net and 3D U-Net. The 3.5Dv4 U-Net combines the predictions of deep learning models trained by 2Da U-Net, 2Dc U-Net, 2Ds U-Net and 3D U-Net. The 3.5Dv5 U-Net combines the predictions of deep learning models trained by 2Da U-Net, 2Dc U-Net, 2Ds U-Net, 2.5D U-Net and 3D U-Net.

In our previous studies, we found the segmentation performance of 2D U-Net varies between different lesions with the DSC ranging from as low as 0.48 in salivary gland tumors^26^ to as high as 0.97 in acute ischemic stroke lesion^25^ on magnetic resonance imaging. In this study, we intentionally employed a total of nine different DLMs to perform automatic segmentation of the teeth. First, three sets of orthogonal images were applied to train axial, coronal, and sagittal 2D U-Nets (named as 2Da U-Net, 2Dc U-Net, and 2Ds U-Net). Second, a 2.5D U-Net was constructed using three continuous axial slices placed in three channels to form an ensemble input image and to train the DLM (named as 2.5D U-Net). Third, a 3D U-Net was constructed using a cuboid (64 × 64 × 128) as an input image. Architectures and hyperparameters of these U-Nets are shown in Table 1. Finally, we applied majority voting to create 4 additional U-Nets. Via combining the predictions of 2D U-Nets trained from each of three orthogonal slices^24^ using majority voting, a 2.5Dv U-Net was generated. Three additional 3.5D U-Nets (i.e., 3.5Dv3 U-Net, 3.5Dv4 U-Net, and 3.5Dv5 U-Net) were generated via majority voting the predictions of 2D U-Nets, 2.5D U-Net, and 3D U-Net at different combination strategies as illustrated in Fig. 2.

**Table 1:** Architectures and hyperparameters of 2D U-Net, 2.5Da U-Net, and 3D U-Net structures.

		2D U-Net	2.5Da U-Net	3D U-Net
Architecture	Convolution	Size = 3 × 3 Stride = 1 Zero-padding	Size = 3 × 3 Stride = 1 Zero-padding	Size = 3 × 3 × 3 Stride = 1 Zero-padding
	Down sampling maxpooling	Size = 2 × 2 Stride = 1	Size = 2 × 2 Stride = 1	Size = 2 × 2 × 2 Stride = 1
	Up sampling	Size = 2 × 2 Stride = 1	Size = 2 × 2 Stride = 1	Size = 2 × 2 × 2 Stride = 1
	Activation function	ReLu	ReLu	ReLu
	U-Net layers	4	4	4
	First layer features	32	32	32
Hyper parameter	Input data size	512 × 512 × 1	512 × 512 × 3	64 × 64 × 128
	Optimizer	Adam	Adam	Adam
	Loss function	BCE	BCE	BCE
	Initial learning rate	0.0001	0.0001	0.0001
	Batch size	12	12	6
	Epoch	150	150	200
Callback function	Reduce learning rate (newLR = LR × 0.95 when val_loss in 10 epochs are no better) Early stopping (training stop when val_loss in 50 epochs are no better)			

*Adam* adaptive moment estimation,* BCE* binary cross entropy, *ReLU* rectified linear unit.

Prediction of each of aforementioned nine U-Nets was treated by basic operations of mathematical morphology, i.e., erosion and dilation. The binary erosion of *I* by *B*, denoted by $I\ominus B$, is defined as Eq. (1):

1 $$I\ominus B=\left\{z\in E|{B}_{z}\subseteq I\right\},$$

where *E* denotes a Euclidean space, *I* denotes a binary image in *E*, *B* denotes a spherical structuring element with a radius of 2 pixels, and *B*_*z*_ denotes the translation of *B* by the vector z. The binary dilation of *I* by *B*, denoted by $I\oplus B$, is defined as Eq. (2):

2 $$I\oplus B=\left\{z\in E|{({B}^{s})}_{z}\cap I\ne \phi \right\},$$

where *B* denotes a spherical structuring element with a radius of 2 pixels, B^s^ denotes the symmetric of B as defined by Eq. (3):

3 $${B}^{s}=\left\{x\in E|-x\in B\right\}$$

### Cross validation and model performance evaluation

**Figure 3:**
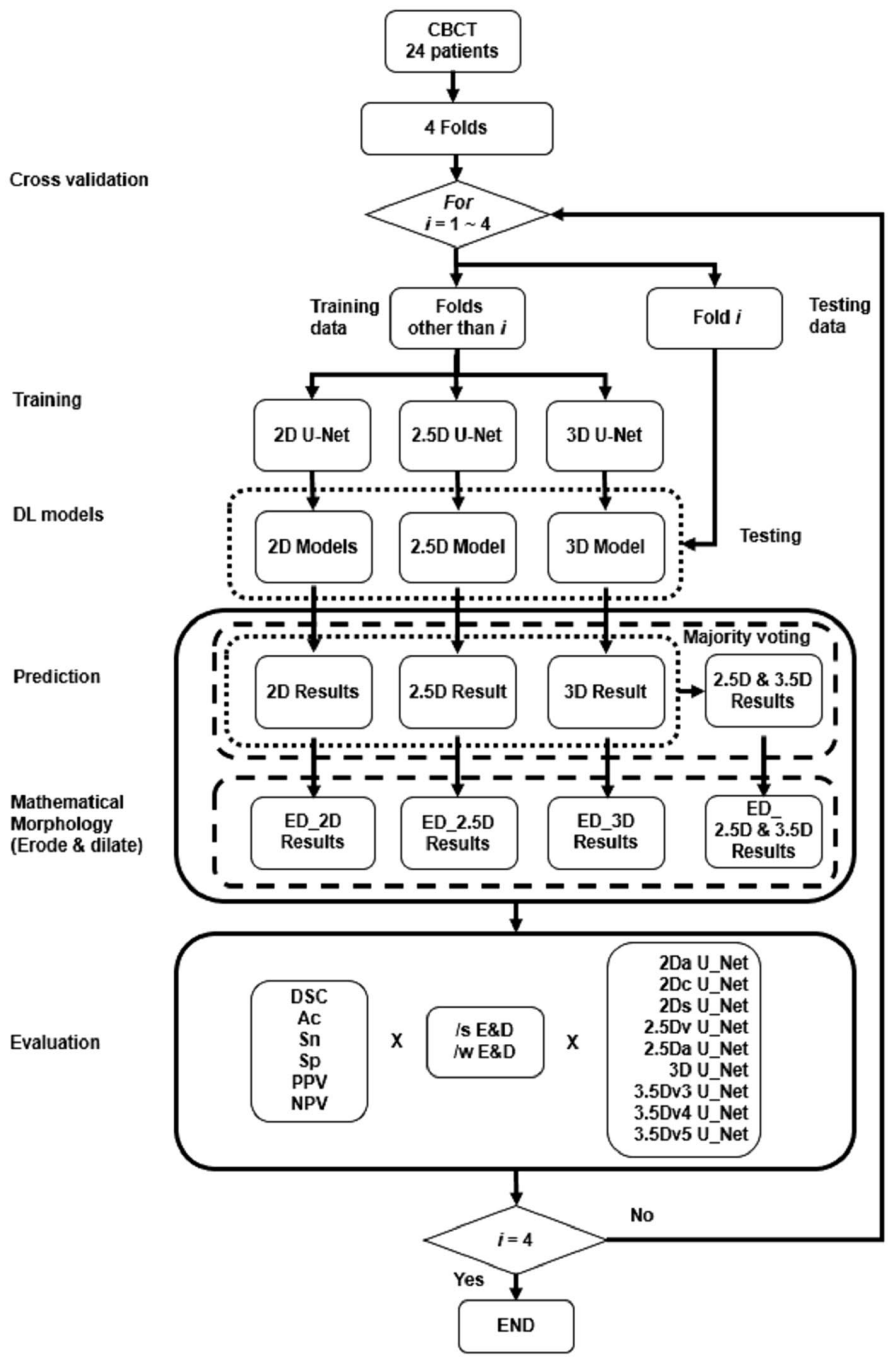
Flowchart of U-Nets in automatic segmentation of teeth using fourfold cross validation.

The flowchart of U-Nets in automatic segmentation of teeth using fourfold cross validation was shown in Fig. 3^27^. Slice-based evaluation of the performance of a DLM was conducted using four-fold cross validation to reflect the performance of a DLM in every slice^28^. The overall segmentation performance was calculated by averaging the performance of every slice^28^. Each voxel of the CBCT image was defined as true positive (TP), true negative (TN), false positive (FP) and false negative (FN) by comparing the prediction to the GT. Segmentation performance of DLMs was evaluated using DSC, Ac, Sn, Sp, PPV, and NPV defined by Eqs. (4) to (9), respectively.

4 $$Ac=\frac{TP+TN}{FP+TP+FN+TN}$$

5 $$DSC=\frac{2TP}{FP+2TP+FN}$$

6 $$Sn=\frac{TP}{TP+FN}$$

7 $$Sp=\frac{TN}{TN+FP}$$

8 $$PPV=\frac{TP}{TP+FP}$$

9 $$NPV=\frac{TN}{TN+FN}$$

### Statistical analysis

In statistical analyses, the normality of data was analyzed first using Kolmogorov–Smirnov test first. Paired Wilcoxon rank test was used to compare continuous data before and after E&D. A nonparametric Kruskal–Wallis test with post hoc analysis using Bonferroni correction was applied for group comparison among 9 U-Nets. A P value less than 0.05 was considered as statistically significant.

## Results

A total of 24 patients were finally recruited, including 15 men and 9 women, with an age of 29.1 ± 14.7 years (mean ± standard deviation). Demographic characteristics of different subsets and groups of patients were summarized in Table 2. There was no difference of age among different subsets of patients (*P* = 0.5658). Impacted teeth were the most common clinical diagnosis, comprising 75% (18 of 24) of patients received CBCT examination.

**Table 2:** Demographics of patients in different subset.

Subset	1	2	3	4	*P* value
**Clinical diagnosis**					
Patient number	6	6	6	6	
Gender (M: F)	3: 3	5: 1	5: 1	2: 4	
Age (years)	25.3 ± 10.6	41.3 ± 22.9	30.1 ± 6.5	19.8 ± 4.2	0.566
Caries	1	1	0	1	
Impacted tooth	5	3	4	6	
Periodontitis	1	2	3	0	
Acute apical periodontitis	1	0	0	0	
Implant design	2	2	2	1	
Residual root	0	0	1	0	

### Comparisons of DSC among U-Nets

**Figure 4:**
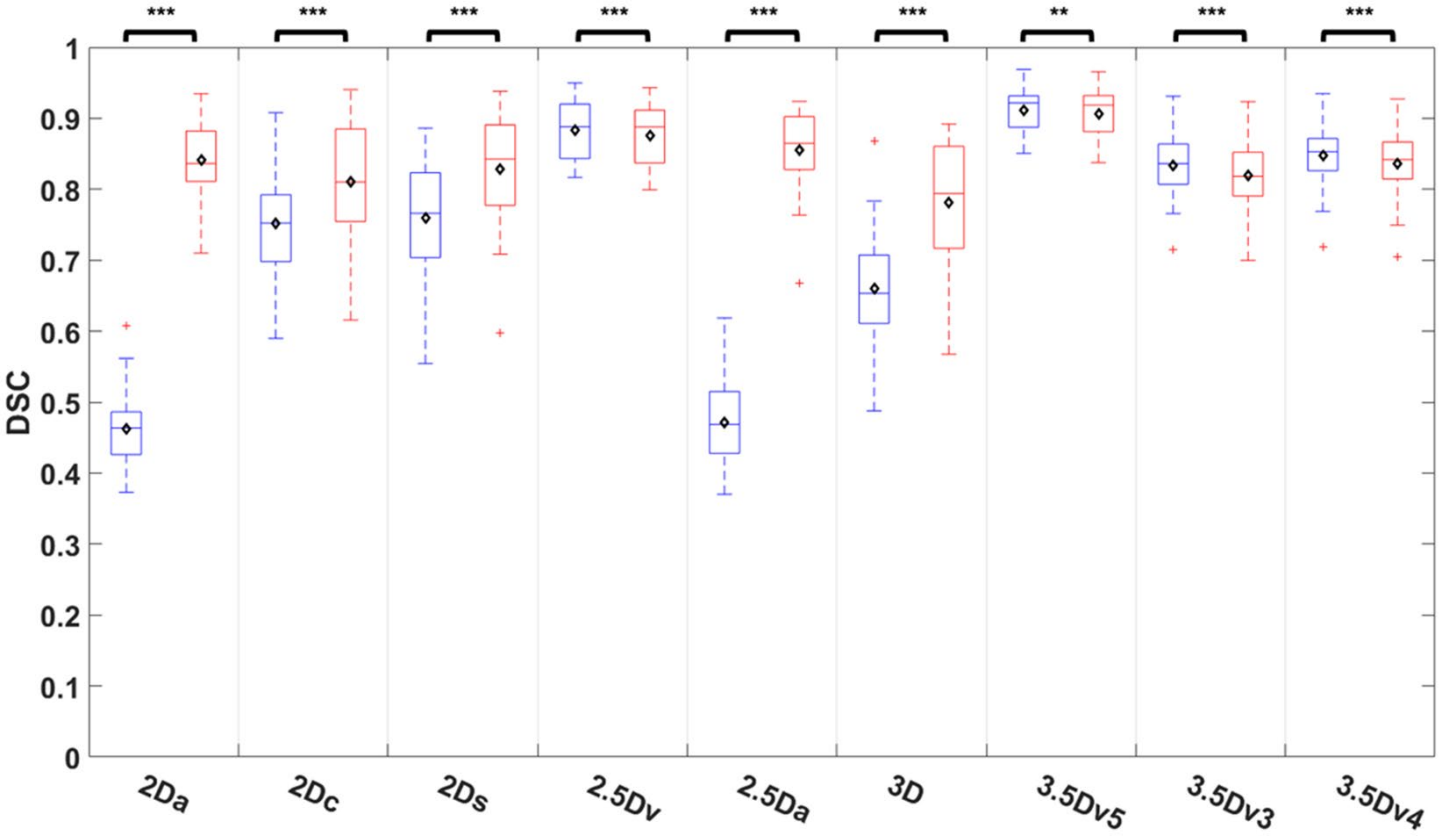
Comparison of DSC among 9 U-Nets before and after E&D.

Comparisons of DSC among nine different U-Nets before and after E&D were shown on Fig. 4 and Table S1. The DSC after E&D was significantly different that before E&D in all U-Nets (all *P* < 0.01). While the DSC after E&D was significantly higher than that before E&D in 5 originally trained U-Nets (all *P* < 0.005), it was significantly lower than that before E&D in 4 U-Nets generated after majority voting (all *P* < 0.01). Before E&D, the 3.5Dv5 U-Net achieved highest DSC which was significantly higher than any of five originally trained U-Nets (all *P* < 0.005), while the 2Da U-Net and 2.5D U-Net performed poorest with DSC significantly lower than other U-Nets (*P* < 0.005) except 3D U-Net (*P* = 0.174 to 0.222). After E&D, the 3.5Dv5 U-Net achieved highest DSC which was significantly higher than most U-Nets (*P* < 0.01) except 2.5Dv U-Net (*P* = 0.551) and 2.5Da U-Net (*P* = 0.07).

### Comparisons of accuracy among U-Nets

**Figure 5:**
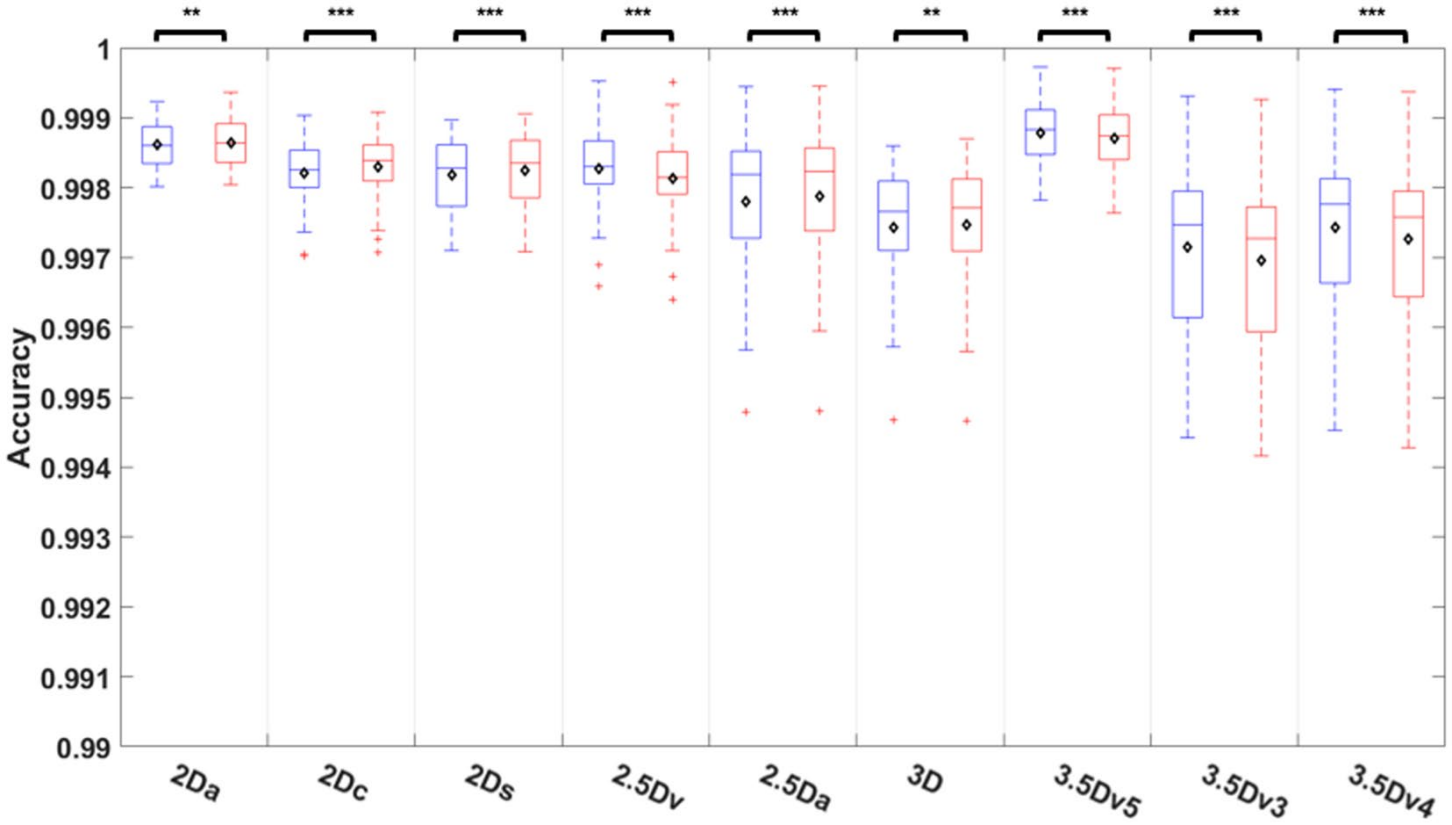
Comparison of accuracy among 9 U-Nets before and after E&D.

Comparisons of accuracy among 9 different U-Nets before and after E&D were shown on Fig. 5 and Table S2. The accuracy after E&D was significantly different that before E&D in all U-Nets (all *P* < 0.01) with the median accuracy higher than 0.997 in all U-Nets no matter before or after E&D. While the accuracy after E&D was significantly higher than that before E&D in 5 originally trained U-Nets (all *P* < 0.01), the it was significantly lower than before E&D in 4 U-Nets generated after majority voting (all *P* < 0.005). Before E&D, the 3.5Dv5 U-Net achieved highest accuracy which was significantly higher than that of 2.5Da U-Net, 3D U-Net, 3.5Dv3 U-Net, and 3.5Dv4 U-Net (*P* < 0.01). After E&D, the 3.5Dv5 U-Net still achieved highest accuracy, which was significantly higher than 2.5Da U-Net, 3D U-Net, 3.5Dv3 U-Net, and 3.5Dv4 U-Net (*P* < 0.05).

### Comparisons of sensitivity among U-Nets

**Figure 6:**
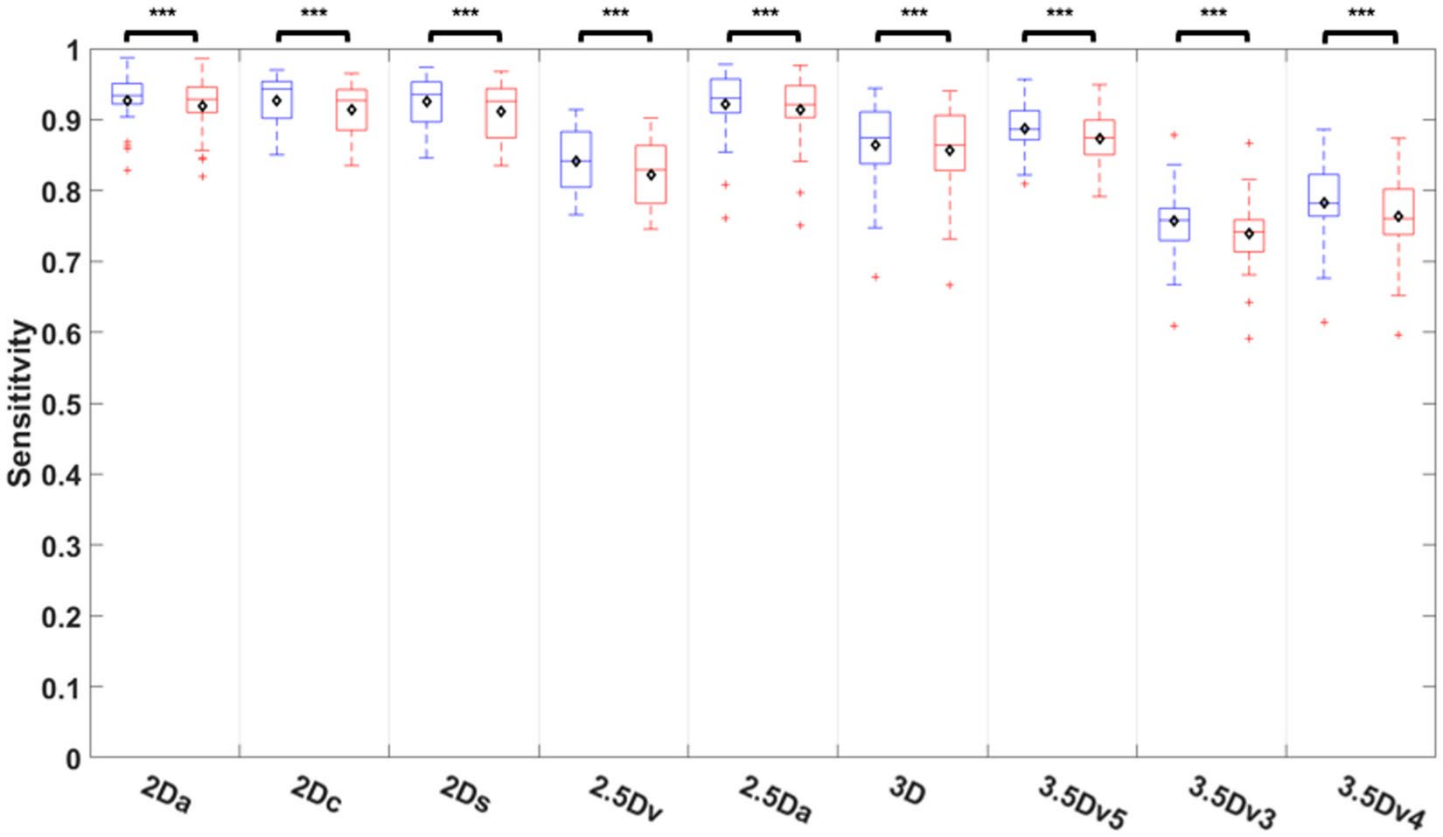
Comparison of sensitivity among 9 U-Nets before and after E&D.

Comparisons of sensitivity among nine different U-Nets before and after E&D was shown on Fig. 6 and Table S3. Before E&D, the 2Dc U-Net achieved highest sensitivity, followed by the 2Ds U-Net, 2Da U-Net, 2.5Da U-Net, and 3.5Dv5 U-Net (*P* = 0.243 to 1), which was significantly higher than that of the 3D U-Net (*P* < 0.05) and other U-Nets with majority voting (*P* < 0.005). E&D significantly reduced the sensitivity in all U-Nets (all *P* < 0.005). After E&D, the 2Da U-Net achieved highest sensitivity, followed by 2Dc U-Net, 2Ds U-Net, 2.5Da U-Net, and 3.5Dv5 U-Net (*P* = 0.141 to 1), which was significantly higher than that of the 3D U-Net (*P* < 0.05) and other U-Nets with majority voting (*P* < 0.005).

### Comparisons of specificity among U-Nets

**Figure 7:**
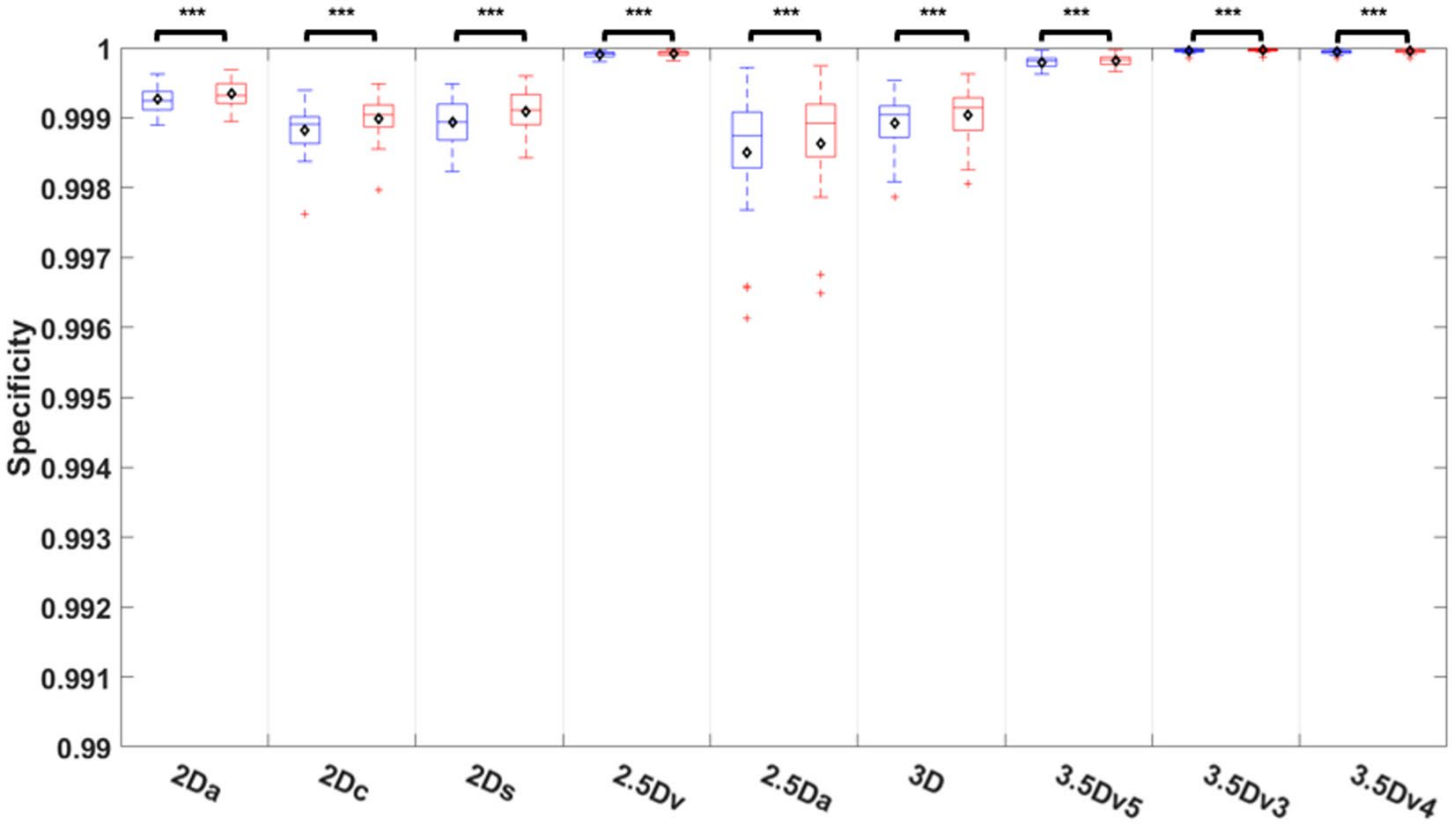
Comparison of specificity among 9 U-Nets before and after E&D.

Comparisons of specificity among nine different U-Nets before and after E&D was shown on Fig. 7 and Table S4. The specificity after E&D was significantly higher than that before E&D in all U-Nets (all *P* < 0.005) with the median specificity higher than 0.998 in all U-Nets before or after E&D. The 3.5Dv3 U-Net and 2.5Dv U-Net achieved a median specificity of 1, significantly higher than that of the 3.5Dv5 U-Net (*P* < 0.05) and all 5 originally trained U-Nets no matter before or after E&D (all *P* < 0.005).

### Comparisons of PPV among U-Nets

**Figure 8:**
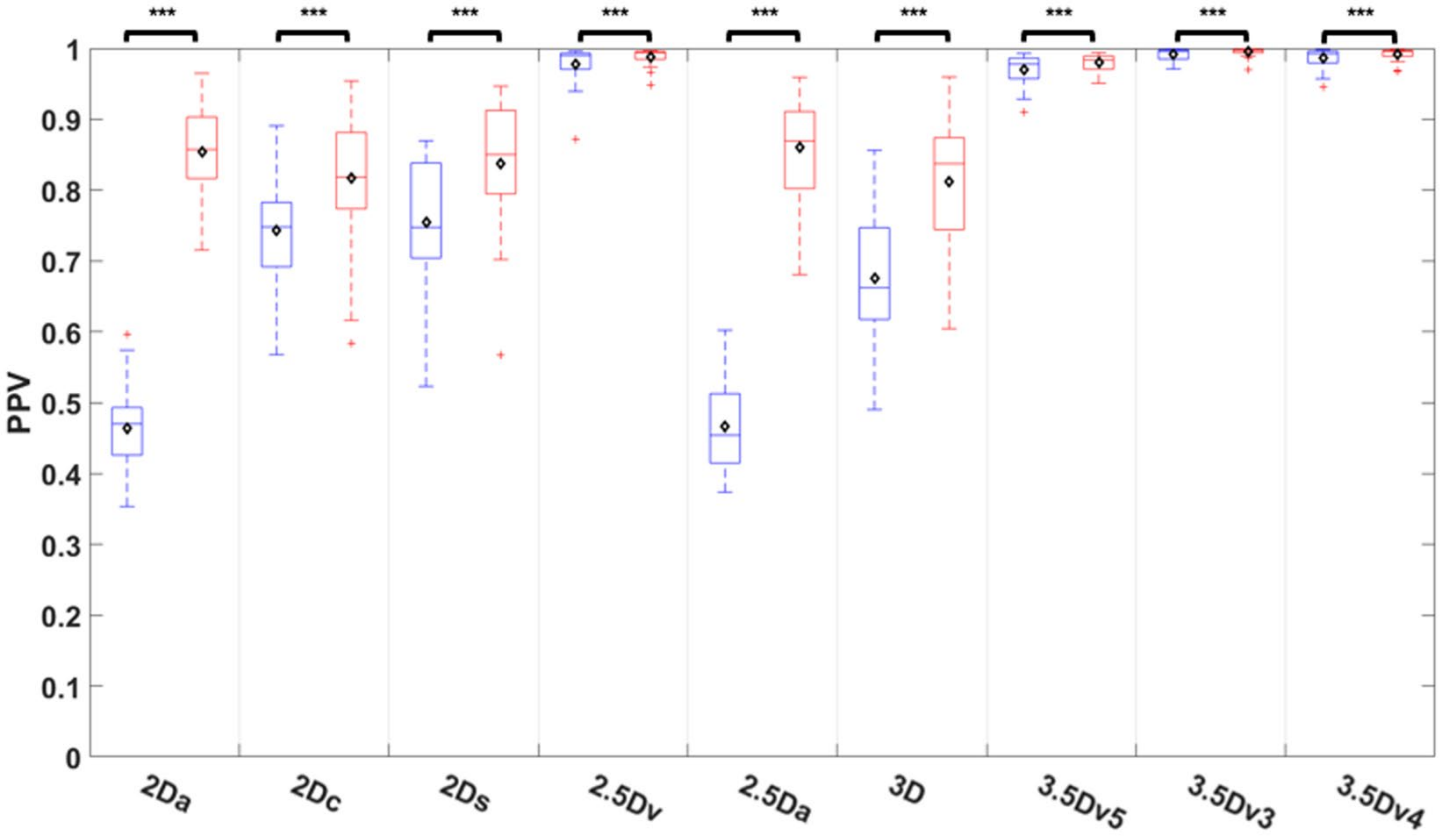
Comparison of positive predict value among 9 U-Nets before and after E&D.

Comparisons of PPV among nine different U-Nets before and after E&D was shown on Fig. 8 and Table S5. The PPV was improved after E&D in all U-Nets (all *P* < 0.005). Before E&D, the 2Da U-Net and 2.5Da U-Net performed poorest with the PPV significantly lower than that of other U-Nets (*P* < 0.05) except the 3D U-Net (*P* = 0.197). The 3.5Dv3 U-Net achieved highest PPV which was similar to the 3.5Dv4 U-Net, 3.5Dv5 U-Net, and 2.5D U-Net (*P* = 0.405 to 0.922) but significantly higher than that of all 5 originally trained U-Nets (all *P* < 0.005). After E&D, the 2Da U-Net and 2.5Da U-Net performed similar to other originally trained U-Nets (*P* = 849 to 1). The 3.5Dv3 U-Net still achieved highest PPV which was similar to the 3.5Dv4 U-Net, 3.5Dv5 U-Net, and 2.5D U-Net (*P* = 0.184 to 0.995) but significantly higher than all 5 originally trained U-Nets (all *P* < 0.005).

### Comparisons of NPV among U-Nets

**Figure 9:**
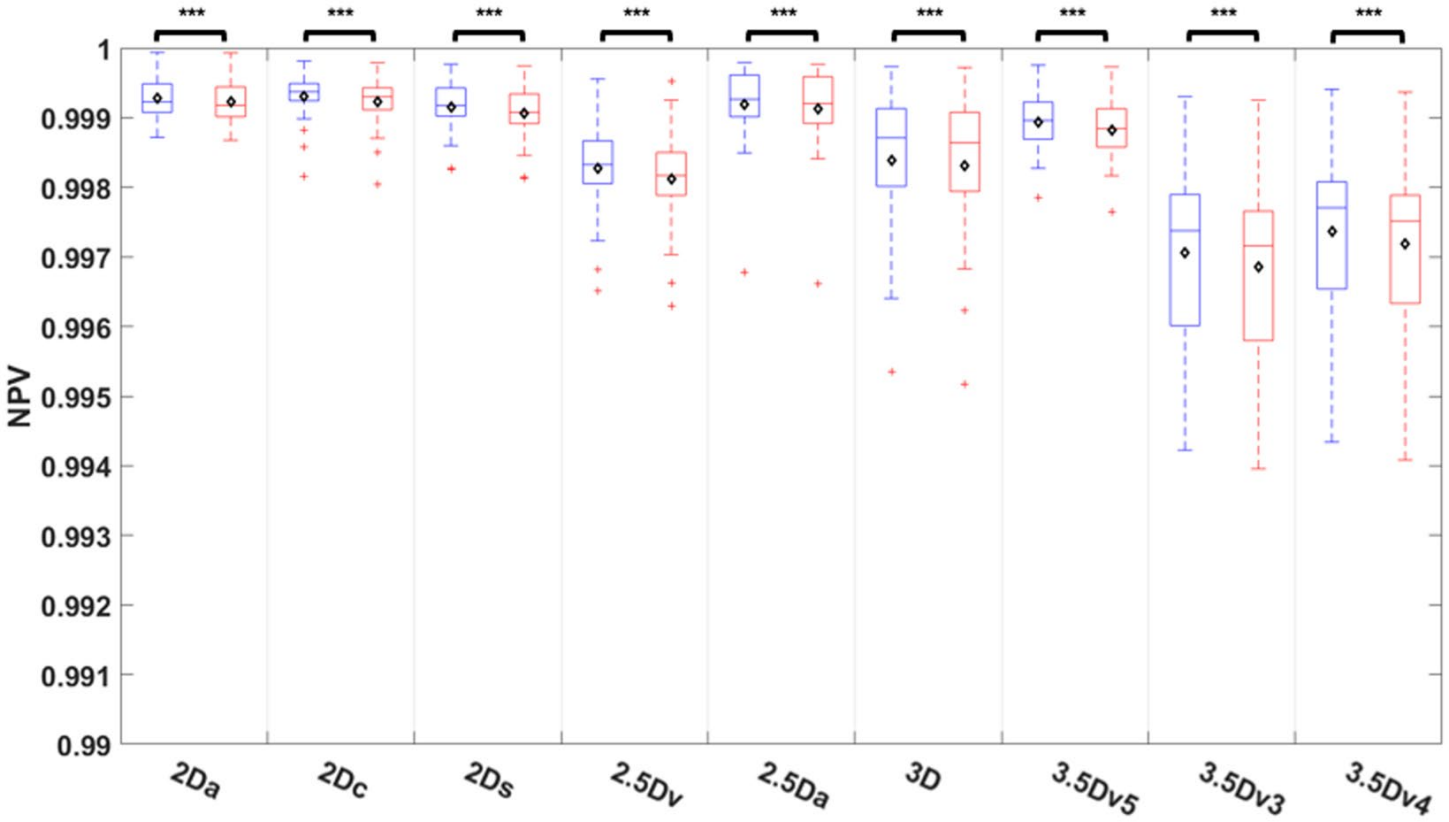
Comparison of negative predict value among 9 U-Nets before and after E&D.

Comparisons of NPV among nine different U-Nets before and after E&D was shown on Fig. 9 and Table S6. E&D significantly reduced the NPV in all U-Nets (all *P* < 0.005) with the median NPV higher than 0.997 in all U-Nets before or after E&D. The 2Dc U-Net achieved highest NPV, followed by 2Da U-Net, 2.5Da U-Net, 2Ds U-Net, and 3.5Dv5 U-Net (*P* = 0.278 to 1), and significantly higher than 3D U-Net (*P* < 0.01) and other U-Nets with majority voting (*P* < 0.005) no matter before or after E&D.

### Case demonstration

**Figure 10:**
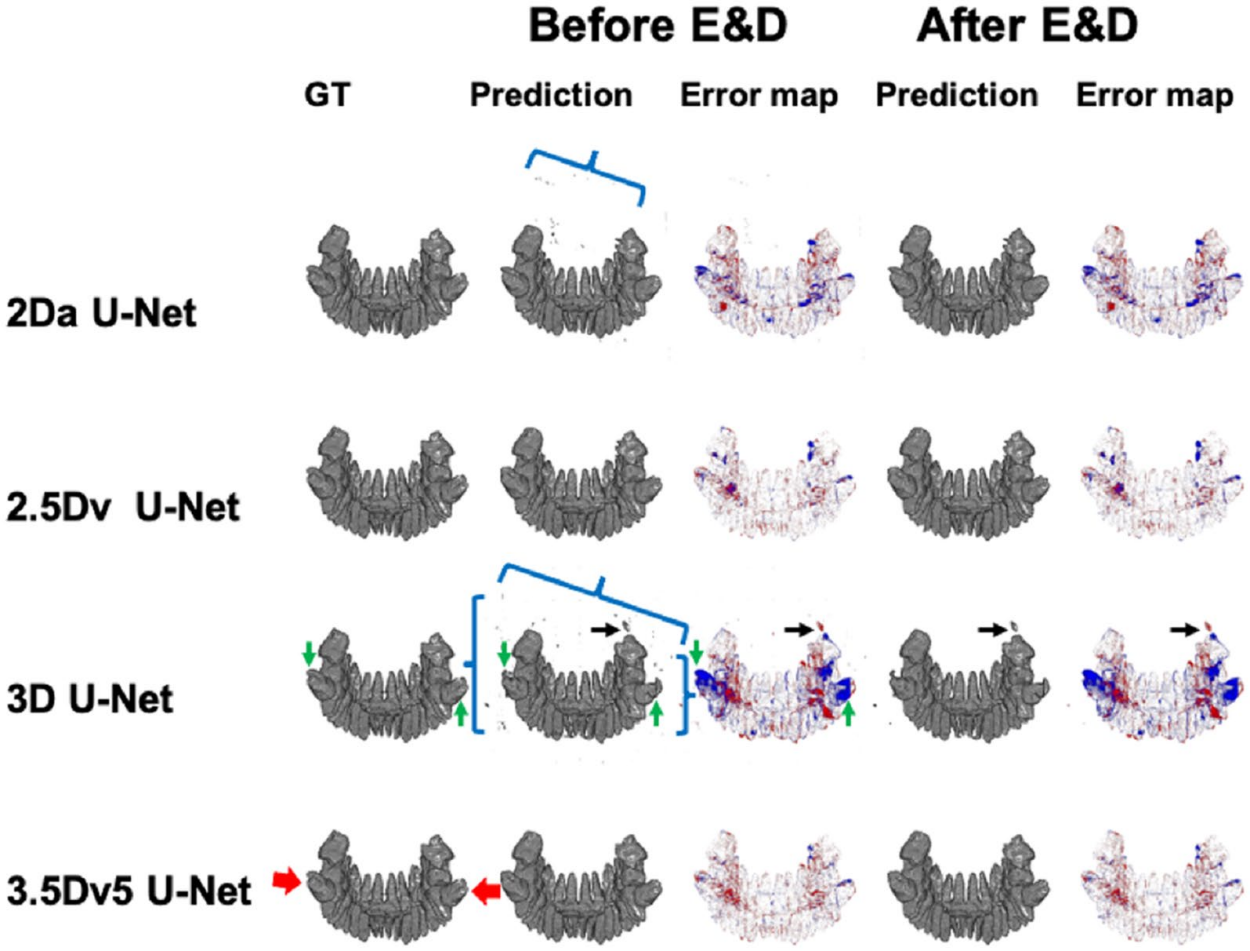
Illustration of ground truth, prediction, and error map in a patient with impacted mandibular third molar teeth (red arrows) before and after E&D in 2Da U-Net, 2.5Dv U-Net, 3D U-Net, and 3.5Dv5 U-Net. The 2Da U-Net and 3D U-Net show lots of tiny false positive results (blue brackets) which could be eliminated by either majority voting or E&D. In 3D U-Net, additional larger false positive results (black arrows), which are not reduced by E&D, are successfully eliminated via majority voting (2.5Dv U-Net and 3.5Dv5 U-Net). Some false negative results (green arrows), which are more apparently seen on 3D U-Net before and after E&D, are successfully remedied via majority voting (2.5Dv U-Net and 3.5Dv5 U-Net).

**Figure 11:**
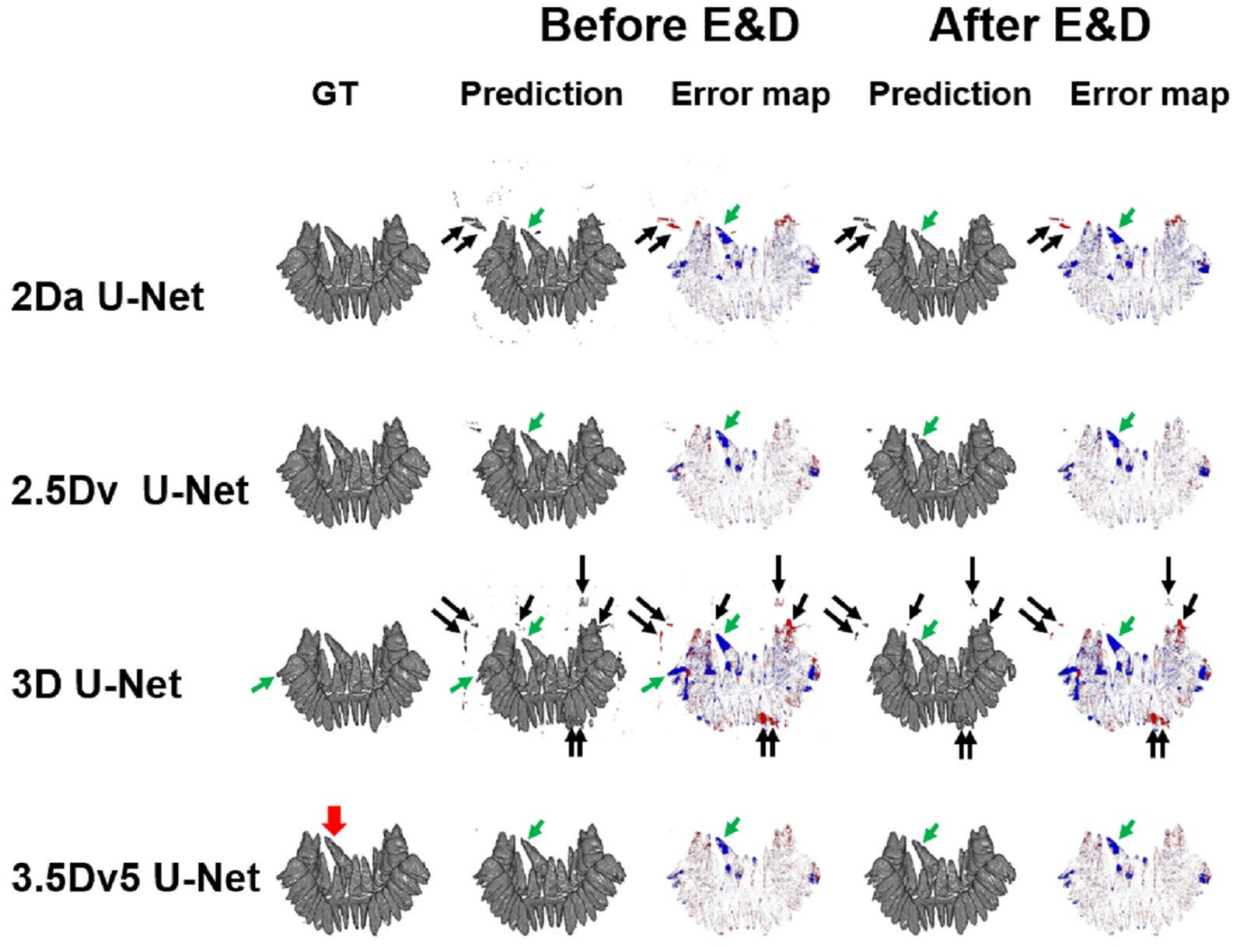
Illustration of ground truth, prediction, and error map in a patient with impacted right maxillary second incisor (red arrow) before and after E&D in 2Da U-Net, 2.5Dv U-Net, 3D U-Net, and 3.5Dv5 U-Net. The 2Da U-Net and 3D U-Net show different false positive results and false negative results, which are eliminated by either majority voting or E&D. Some larger false positive results (black arrows) in the 2Da U-Net and 3D U-Net, which are not eliminated by E&D, are completely eliminated by the 3.5Dv5 U-Net. On the other hand, the false negative results (green arrows) appearing in the 2Da U-Net and the 3D U-Net remain similar on 2.5Dv U-Net and 3.5Dv5 U-Net before and after E&D.

Figures 10 and 11 demonstrate the 3D illustration of predictions and error maps of 4 different U-Nets before and after E&D in two patients.

## Discussion

Accurate segmentation of bony structures and teeth on CBCT is an important foundation of stomatology. Training strategy has been shown to be a factor influencing the segmentation performance of convolutional neural network (CNN) for bony structures on CBCT^24^. In our study, we intentionally applied nine different training strategies based on the U-Net architecture and compared the performance in teeth segmentation on CBCT among different strategies. Our study demonstrated that the segmentation performance of the U-Net varied among different training strategies. The 2Da U-Net and the 2.5Da U-Net had poor segmentation performance with a median DSC of 0.464 and 0.469, respectively. The segmentation performance of the 2Da U-Net was improved via 3 strategies. First, by changing the input imaging data, the median DSC was significantly improved to 0.752 and 0.766 in the 2Dc U-Net and the 2Ds U-Net, respectively (via changing slice orientation) and slightly improved to 0.653 in the 3D U-Net (via supplying additional z-axis information). Second, by using majority voting, the median DSC was significantly improved to 0.922 (3.5Dv5 U-Net). Third, by employing mathematical morphology using E&D, the median DSC was significantly improved to 0.836 and 0.865 in the 2Da U-Net and the 2.5Da U-Net, respectively. Table 3 compares the segmentation performance of our proposed methods to those proposed by other researchers. The DSC in our study is relatively lower than some previous studies^20,21,27,29–32^, in which the DSC ranges from 0.934^31^ to 0.97^30^. In our study, we calculated the DSC slice-by-slice and then averaged the DSC of all slices rather than calculated the DSC for the whole CBCT volume as other studies^20,21,23,27,29–31,33–37^. Nevertheless, the highest DSC achieved by our 3.5Dv5 U-Net is consistent with other previous studies^23,33–35^, in which the DSC ranges from 0.9^23^ to 0.921^33^. Our study achieved an accuracy ranging from 0.997 to 0.999 which is higher than that reported in previous studies^30,36,37^. Our 2D U-Nets achieved a sensitivity ranging from 0.934 to 0.943 which is similar to that (0.91 to 0.94 and 0.932) of Fontenele’s study^30^ and Lee’s study^34^, respectively, and higher than that (0.83) of Shaheen’s study^23^. In addition, our U-Nets with majority voting achieve a PPV ranging from 0.978 to 0.996 which is similar to that (0.98) of Shaheen’s study^23^ and higher than that (0.904) of Lee’s study^34^.

**Table 3:** Comparison of segmentation of human teeth on CBCT using CNN.

Author	Year	Patients/images	CNN architecture	Training strategy	Evaluation strategy	DSC	Ac	Sn	SP	PPV	NPV
Xu^37^	2019	1200/NA	DNN	3D volume	VB	NA	0.991	NA	NA	NA	NA
Tian^36^	2019	600/NA	U-Net + HN	3D volume	VB	NA	0.898	NA	NA	NA	NA
Cui^33^	2019	20/NA	ToothNet	3D volume	VB	0.921	NA	NA	NA	NA	NA
Li^21^	2020	24/1160	AttU-Net + BDC-lstm	2D slices	VB	0.9526	NA	NA	NA	NA	NA
Lee^34^	2020	102/NA	UDS-Net	2D slices	NA	0.918	NA	0.932	NA	0.904	NA
Chen^29^	2020	25/NA	FCN + MWT	3D volume	NA	0.936	NA	NA	NA	NA	NA
Rao^35^	2020	NA/86	SFCRN + DCRF	2D slices	NA	0.917	NA	NA	NA	NA	NA
Wu^32^	2020	20/NA	GH + BADice + DASPP U-Net	3D volume	VB	0.962	NA	NA	NA	NA	NA
Wang^27^	2021	28/9507	MS-D	NA	VB	0.945	NA	NA	NA	NA	NA
Duan^20^	2021	30/NA	U-Net	2D slices	VB	0.957	NA	NA	NA	NA	NA
Shaheen^23^	2021	186/NA	3D U-Net	3D volume	VB	0.90^†^	NA	0.83	NA	0.98	NA
Lahoud^31^	2021	314/2924	FPN	2D slices	VB	0.934	NA	NA	NA	NA	NA
Fontenele^30^	2022	175/	3D U-Net	3D volume	VB	0.95–0.97	0.994–0.997	0.91–0.94	NA	1	NA
Our study	2022	24/12,552	2Da U-Net	2D slices	SB	0.839^a^	0.999	0.925	0.999	0.852^a^	0.999
			3D U-Net	3D volume	SB	0.779^a^	0.997	0.864	0.999	0.810^a^	0.998
			3.5Dv5 U-Net	2D slices, 3D volume	SB	0.911	0.999	0.888	1	0.970	0.999

Numerical data are presented as mean value.

*BADice* boundary aware dice loss, *BDC-LSTM* bidirectional convolution long short-term memory, *DASPP* densely connected Atrous spatial pyramid pooling, *DCRF* dense conditional random field, *FCN* fully convolutional network, *FPN* feature pyramid network, *GH* Gaussian heatmap localization, *HN* hierarchical network, *LO* label optimization, *MS-D* mixed-scale dense, *MWT* marker-controlled watershed transform, *NA* not available, *PB* volume-based, *SB* slice-based, *SFCRN* symmetric fully convolutional residual network, *UDS-Net* U-Net added by dense block and spatial dropout.

^a^Data acquired after erosion and dilation of mathematical morphology.

Segmentation of teeth on whole volume of CBCT remains challenging on 2D U-Net because of the similar Hounsfield units between teeth and bony structures and insufficient spatial information along the perpendicular direction for the input images, i.e., lacking z-axis information in axial slice, y-axis information in coronal slice, and x-axis information in sagittal slice. Solely using axial images as input data, 2Da U-Net tends to predict clusters of tooth root-mimicking bony structures on axial plane false positively. Based on the Eq. (5), the DSC of a slice with any pixel which was predicted as tooth but were out of range of teeth in GT was zero. Accordingly, the overall DSC dropped due to the false positive results of prediction on slices that do not contain any pixel of teeth on GT. These false positive results on 2Da U-Net have two characteristic features, including (1) no specific spatial connection between two clusters along the z-axis and (2) specific tooth root-mimicking geometric shapes, i.e., round or ovoid shapes. Such false positive results could be eliminated or reduced by changing the orientation of the input slices from axial to coronal or sagittal. By choosing coronal slices or sagittal slices as input, 2Dc U-Net and 2Ds U-Net provided abundant z-axis information for the model to recognize the connection of tooth roots and the whole tooth and therefore help eliminate parts of false positive results around the tooth roots. Although the small round or ovoid false positive results on 2Da U-Net were reduced, 2Dc U-Net and 2Ds U-Net had drawbacks by taking the sheet-like bony structures as teeth false positively. The false positive results on 2Da U-Net could also be remedied by providing additional z-axis information in a 3D patch as input data. However, the 3D U-Net produced some different false positive results while reducing those on 2Da U-Net. These false positive results might be attributed to the insufficient and discontinuous information at the edge of each 3D patch.

Majority voting has been used to improve the segmentation performance of anatomic structures on MR images^38^, conventional CT images^39^, and CBCT^24,40^ by combing the prediction from axial, coronal, and sagittal images. We intentionally applied different voting strategies from five original U-Nets (i.e., 2Da U-Net, 2Dc U-Net, 2Ds U-Net, 2.5Da U-Net, and 3D U-Net) to generate 4 additional virtual U-Nets (i.e., 2.5Dv U-Net, 3.5Dv5 U-Net, 3.5Dv4 U-Net, 3.5Dv3 U-Net) in order to compare the performance of different weighting of majority voting. The 2.5Dv U-Net integrated results from three 2D U-Nets (2Da U-Net, 2Dc U-Net, and 2Ds U-Net) as used in prior studies^24,38,39^, while the 3.5D U-Nets integrate these 2D U-Nets together with additional 2.5Da U-Net and 3D U-Net. Our results show that the U-Nets with majority voting (2.5Dv U-Net, 3.5Dv3 U-Net, and 3.5Dv5 U-Net) improved segmentation performance with DSC significantly higher than originally trained U-Nets. By integrating five originally trained U-Nets, the 3.5Dv5 U-Net showed highest DSC, accuracy, specificity, and NPV.

Diminutive noise speckles could be eliminated using mathematical morphology^41^. The combination of erode and dilate operators is capable of noise removal by eroding the image with a kernel followed by dilating the image with another kernel. By applying 3D erosion and dilation, our results showed significant changes in segmentation performance, including significantly higher specificity and PPV of all U-Nets, significantly higher DSC and accuracy of all originally trained U-Nets but significantly lower DSC and accuracy of all U-Nets with majority voting, but significantly lower sensitivity and NPV in all U-Nets.

Our study has some limitations to be addressed. First, the sample size of our study is relatively small. Our sample size is similar to that in Li’s study (N = 24), Chen’s study (N = 25)^29^, Wu’s study (N = 20)^32^, Wang’s study (N = 28)^27^, and Duan’s study (N = 30)^20^. To remedy it, we applied fourfold cross validation to verify our results. Second, the GT was not purely defined by senior dentists but by a third-year resident in periodontology and 3 different junior researchers, leading potential bias in defining the GT of teeth. To remedy it, all GTs were slice-by-slice verified and corrected by a senior neuroradiologist. Third, we did not evaluate interobserver agreement and intraobserver reliability in this study. Further study designed to evaluate the interobserver agreement and intraobserver reliability is warranted to reduce the potential bias occurring in the step of GT generation. Fourth, we did not perform apply any boning box for the teeth in our study. We intentionally used whole volume of CBCT to train and test all U-Nets to compare the segmentation performance of U-Nets with different training strategies not only in the teeth-containing slices but also in slices beyond the levels of teeth. Finally, we did not calculate the volume-based performance matrix as previous studies. By using slice-based performance matrix, our study clearly discloses the pros and cons of different training strategies of U-Nets on the one hand and also allows comparison between our results and others’ results on the other hand. Finally, we did not evaluate the diagnostic performance of the proposed method in any specific dental pathologies although the majority (75%) of patients received CBCT examination in order to evaluate the details of impacted teeth. To evaluate the diagnostic performance of the proposed 3.5D U-Net, further study enrolling specific dental pathology is warranted.

## Conclusion

Performance of U-Nets varies among different training strategies for teeth segmentation on CBCT. The segmentation performance of the U-Net can be improved by majority voting and E&D. Overall speaking, the 3.5Dv5 U-Net achieved the best segmentation performance among all U-Nets.

## Supplementary Information

Supplementary Tables.

## Data Availability

The datasets used or analyzed during the current study are available from the corresponding author on reasonable request.

## Abbreviations

2D U-Net: U-Net using a 2D image as the unit of the input data
2Da U-Net: U-Net using an axial slice as the unit of the input data
2Dc U-Net: U-Net using a coronal slice as the unit of the input data
2Ds U-Net: U-Net using a sagittal slice as the unit of the input data
2.5Da U-Net: U-Net using three continuous axial slices as the unit of the input data
2.5Dv U-Net: U-Net integrating the predictions of 2Da U-Net, 2Dc U-Net, and 2Ds U-Net via majority voting
3D U-Net: U-Net using a cuboid as the unit of the input data
3.5Dv3 U-Net: U-Net integrating the predictions of 2.5Dv U-Net, 2.5Da U-Net, and 3D U-Net via majority voting
3.5Dv4 U-Net: U-Net integrating the predictions of 2Da U-Net, 2Dc U-Net, 2Ds U-Net, and 3D U-Net via majority voting
3.5Dv5 U-Net: U-Net integrating the predictions of 2Da U-Net, 2Dc U-Net, 2Ds U-Net2.5Da U-Net, and 3D U-Net via majority voting
Ac: Accuracy
CBCT: Cone beam computed tomography
DLM: Deep learning model
DSC: Dice similarity coefficient
E&D: Erosion and dilation
FN: False negative
FP: False positive
GT: Ground truth
HMDB: Heavy metallic dental burden
NPV: Negative predictive value
PPV: Positive predictive value
Sn: Sensitivity
Sp: Specificity
TN: True negative
TP: True positive
