# Supplementary material for: Improving performance of deep learning models using 3.5D U-Net via majority voting for tooth segmentation on cone beam computed tomography
Source: Sci Rep. 2022 Nov 17;12:19809. doi: 10.1038/s41598-022-23901-7 (PMC9672125; doi:10.1038/s41598-022-23901-7)
Supplement: Supplementary file 1 — Supplementary Tables. [file 41598_2022_23901_MOESM1_ESM.docx]

Supplementary materials

Table S1. Statistical analysis of Dice similarity coefficient (DSC) among 9 U-Nets using Kruskal-Wallis test with post hoc analysis using Bonferroni correction before and after E&D.

| **DSC** | | **After E&D** | | | | | | | | |  |
| --- | --- | --- | --- | --- | --- | --- | --- | --- | --- | --- | --- |
|  |  | **2Da** | **2Dc** | **2Ds** | **2.5Dv** | **2.5Da** | **3D** | **3.5Dv5** | **3.5Dv3** | **3.5Dv4** |  |
| **Before E&D** | **2Da** |  | 0.994 | 1.000 | 0.542 | 0.986 | 0.592 | ** | 0.939 | 1.000 | **2Da** |
|  | **2Dc** | *** |  | 0.998 | 0.092 | 0.623 | 0.981 | *** | 1.000 | 0.999 | **2Dc** |
|  | **2Ds** | *** | 1.000 |  | 0.439 | 0.966 | 0.694 | ** | 0.970 | 1.000 | **2Ds** |
|  | **2.5Dv** | *** | ** | ** |  | 0.986 | ** | 0.551 | * | 0.387 | **2.5Dv** |
|  | **2.5Da** | 1.000 | ** | *** | *** |  | 0.080 | 0.070 | 0.355 | 0.950 | **2.5Da** |
|  | **3D** | 0.174 | 0.805 | 0.650 | *** | 0.222 |  | *** | 0.999 | 0.744 | **3D** |
|  | **3.5Dv5** | *** | *** | *** | 0.961 | *** | *** |  | *** | *** | **3.5Dv5** |
|  | **3.5Dv3** | *** | 0.538 | 0.709 | 0.633 | *** | ** | 0.058 |  | 0.981 | **3.5Dv3** |
|  | **3.5Dv4** | *** | 0.168 | 0.285 | 0.949 | *** | *** | 0.275 | 0.999 |  | **3.5Dv4** |
|  |  | **2Da** | **2Dc** | **2Ds** | **2.5Dv** | **2.5Da** | **3D** | **3.5Dv5** | **3.5Dv3** | **3.5Dv4** |  |

Note: A *P* value no less than 0.05 is shown as numerical data. *, **, and *** denotes a *P* value less than 0.05, 0.01, and 0.005. DSC denotes Dice similarity coefficient. E&D denotes erosion and dilation. 2Da, 2Dc, 2Ds, 2.5Dv, 2.5Da, 3D, 3.5Dv5, 3.5Dv3, and 3.5Dv4 denotes 2Da U-Net, 2Dc U-Net, 2Ds U-Net, 2.5Dv U-Net, 2.5Da U-Net, 3D U-Net, 3.5Dv5 U-Net, 3.5Dv3 U-Net, and 3.5Dv4 U-Net, respectively.

Table S2. Statistical analysis of accuracy among 9 U-Nets using Kruskal-Wallis test with post hoc analysis using Bonferroni correction before and after E&D.

| **Accuracy** | | **After E&D** | | | | | | | | |  |
| --- | --- | --- | --- | --- | --- | --- | --- | --- | --- | --- | --- |
|  |  | **2Da** | **2Dc** | **2Ds** | **2.5Dv** | **2.5Da** | **3D** | **3.5Dv5** | **3.5Dv3** | **3.5Dv4** |  |
| **Before E&D** | **2Da** |  | 0.644 | 0.491 | 0.169 | 0.050 | *** | 1.000 | *** | *** | **2Da** |
|  | **2Dc** | 0.406 |  | 1.000 | 0.997 | 0.947 | 0.077 | 0.524 | ** | * | **2Dc** |
|  | **2Ds** | 0.324 | 1.000 |  | 1.000 | 0.983 | 0.136 | 0.376 | ** | 0.060 | **2Ds** |
|  | **2.5Dv** | 0.672 | 1.000 | 1.000 |  | 1.000 | 0.428 | 0.111 | * | 0.246 | **2.5Dv** |
|  | **2.5Da** | * | 0.982 | 0.993 | 0.890 |  | 0.743 | * | 0.118 | 0.535 | **2.5Da** |
|  | **3D** | *** | 0.132 | 0.180 | * | 0.741 |  | *** | 0.977 | 1.000 | **3D** |
|  | **3.5Dv5** | 0.999 | 0.091 | 0.063 | 0.229 | ** | *** |  | *** | *** | **3.5Dv5** |
|  | **3.5Dv3** | *** | * | 0.057 | * | 0.439 | 1.000 | *** |  | 0.997 | **3.5Dv3** |
|  | **3.5Dv4** | *** | 0.294 | 0.373 | 0.126 | 0.918 | 1.000 | *** | 0.997 |  | **3.5Dv4** |
|  |  | **2Da** | **2Dc** | **2Ds** | **2.5Dv** | **2.5Da** | **3D** | **3.5Dv5** | **3.5Dv3** | **3.5Dv4** |  |

Note: A *P* value no less than 0.05 is shown as numerical data. *, **, and *** denotes a *P* value less than 0.05, 0.01, and 0.005. DSC denotes Dice similarity coefficient. E&D denotes erosion and dilation. 2Da, 2Dc, 2Ds, 2.5Dv, 2.5Da, 3D, 3.5Dv5, 3.5Dv3, and 3.5Dv4 denotes 2Da U-Net, 2Dc U-Net, 2Ds U-Net, 2.5Dv U-Net, 2.5Da U-Net, 3D U-Net, 3.5Dv5 U-Net, 3.5Dv3 U-Net, and 3.5Dv4 U-Net, respectively.

Table S3. Statistical analysis of sensitivity among 9 U-Nets using Kruskal-Wallis test with post hoc analysis using Bonferroni correction before and after E&D.

| **Sensitivity** | | **After E&D** | | | | | | | | |  |
| --- | --- | --- | --- | --- | --- | --- | --- | --- | --- | --- | --- |
|  |  | **2Da** | **2Dc** | **2Ds** | **2.5Dv** | **2.5Da** | **3D** | **3.5Dv5** | **3.5Dv3** | **3.5Dv4** |  |
| **Before E&D** | **2Da** |  | 1.000 | 1.000 | *** | 1.000 | * | 0.141 | *** | *** | **2Da** |
|  | **2Dc** | 1.000 |  | 1.000 | *** | 1.000 | 0.079 | 0.279 | *** | *** | **2Dc** |
|  | **2Ds** | 1.000 | 1.000 |  | *** | 1.000 | 0.109 | 0.347 | *** | *** | **2Ds** |
|  | **2.5Dv** | *** | *** | *** |  | *** | 0.786 | 0.428 | 0.218 | 0.677 | **2.5Dv** |
|  | **2.5Da** | 1.000 | 1.000 | 1.000 | *** |  | 0.075 | 0.266 | *** | *** | **2.5Da** |
|  | **3D** | * | * | * | 0.943 | * |  | 1.000 | ** | * | **3D** |
|  | **3.5Dv5** | 0.243 | 0.230 | 0.294 | 0.459 | 0.339 | 0.995 |  | *** | ** | **3.5Dv5** |
|  | **3.5Dv3** | *** | *** | *** | 0.170 | *** | ** | *** |  | 0.999 | **3.5Dv3** |
|  | **3.5Dv4** | *** | *** | *** | 0.625 | *** | * | ** | 0.998 |  | **3.5Dv4** |
|  |  | **2Da** | **2Dc** | **2Ds** | **2.5Dv** | **2.5Da** | **3D** | **3.5Dv5** | **3.5Dv3** | **3.5Dv4** |  |

Note: A *P* value no less than 0.05 is shown as numerical data. *, **, and *** denotes a *P* value less than 0.05, 0.01, and 0.005. DSC denotes Dice similarity coefficient. E&D denotes erosion and dilation. 2Da, 2Dc, 2Ds, 2.5Dv, 2.5Da, 3D, 3.5Dv5, 3.5Dv3, and 3.5Dv4 denotes 2Da U-Net, 2Dc U-Net, 2Ds U-Net, 2.5Dv U-Net, 2.5Da U-Net, 3D U-Net, 3.5Dv5 U-Net, 3.5Dv3 U-Net, and 3.5Dv4 U-Net, respectively.

Table S4. Statistical analysis of specificity among 9 U-Nets using Kruskal-Wallis test with post hoc analysis using Bonferroni correction before and after E&D.

| **Specificity** | | **After E&D** | | | | | | | | |  |
| --- | --- | --- | --- | --- | --- | --- | --- | --- | --- | --- | --- |
|  |  | **2Da** | **2Dc** | **2Ds** | **2.5Dv** | **2.5Da** | **3D** | **3.5Dv5** | **3.5Dv3** | **3.5Dv4** |  |
| **Before E&D** | **2Da** |  | 0.422 | 0.838 | ** | 0.195 | 0.801 | 0.176 | *** | *** | **2Da** |
|  | **2Dc** | 0.257 |  | 0.999 | *** | 1.000 | 1.000 | *** | *** | *** | **2Dc** |
|  | **2Ds** | 0.642 | 1.000 |  | *** | 0.982 | 1.000 | ** | *** | *** | **2Ds** |
|  | **2.5Dv** | ** | *** | *** |  | *** | *** | 0.842 | 0.812 | 0.992 | **2.5Dv** |
|  | **2.5Da** | 0.166 | 1.000 | 0.997 | *** |  | 0.988 | *** | *** | *** | **2.5Da** |
|  | **3D** | 0.754 | 0.998 | 1.000 | *** | 0.989 |  | *** | *** | *** | **3D** |
|  | **3.5Dv5** | 0.247 | *** | *** | 0.862 | *** | ** |  | * | 0.263 | **3.5Dv5** |
|  | **3.5Dv3** | *** | *** | *** | 0.767 | *** | *** | * |  | 0.999 | **3.5Dv3** |
|  | **3.5Dv4** | *** | *** | *** | 0.986 | *** | *** | 0.238 | 0.999 |  | **3.5Dv4** |
|  |  | **2Da** | **2Dc** | **2Ds** | **2.5Dv** | **2.5Da** | **3D** | **3.5Dv5** | **3.5Dv3** | **3.5Dv4** |  |

Note: A *P* value no less than 0.05 is shown as numerical data. *, **, and *** denotes a *P* value less than 0.05, 0.01, and 0.005. DSC denotes Dice similarity coefficient. E&D denotes erosion and dilation. 2Da, 2Dc, 2Ds, 2.5Dv, 2.5Da, 3D, 3.5Dv5, 3.5Dv3, and 3.5Dv4 denotes 2Da U-Net, 2Dc U-Net, 2Ds U-Net, 2.5Dv U-Net, 2.5Da U-Net, 3D U-Net, 3.5Dv5 U-Net, 3.5Dv3 U-Net, and 3.5Dv4 U-Net, respectively.

Table S5. Statistical analysis of positive predictive value (PPV) among 9 U-Nets using Kruskal-Wallis test with post hoc analysis using Bonferroni correction before and after E&D.

| **PPV** | | **After E&D** | | | | | | | | |  |
| --- | --- | --- | --- | --- | --- | --- | --- | --- | --- | --- | --- |
|  |  | **2Da** | **2Dc** | **2Ds** | **2.5Dv** | **2.5Da** | **3D** | **3.5Dv5** | **3.5Dv3** | **3.5Dv4** |  |
| **Before E&D** | **2Da** |  | 0.999 | 1.000 | *** | 1.000 | 0.997 | *** | *** | *** | **2Da** |
|  | **2Dc** | * |  | 1.000 | *** | 0.993 | 1.000 | *** | *** | *** | **2Dc** |
|  | **2Ds** | ** | 1.000 |  | *** | 1.000 | 1.000 | *** | *** | *** | **2Ds** |
|  | **2.5Dv** | *** | ** | ** |  | *** | *** | 0.976 | 0.849 | 0.999 | **2.5Dv** |
|  | **2.5Da** | 1.000 | * | ** | *** |  | 0.989 | ** | *** | *** | **2.5Da** |
|  | **3D** | 0.197 | 0.991 | 0.981 | *** | 0.189 |  | *** | *** | *** | **3D** |
|  | **3.5Dv5** | *** | * | * | 0.995 | *** | *** |  | 0.184 | 0.712 | **3.5Dv5** |
|  | **3.5Dv3** | *** | *** | *** | 0.922 | *** | *** | 0.405 |  | 0.995 | **3.5Dv3** |
|  | **3.5Dv4** | *** | *** | *** | 0.999 | *** | *** | 0.828 | 0.999 |  | **3.5Dv4** |
|  |  | **2Da** | **2Dc** | **2Ds** | **2.5Dv** | **2.5Da** | **3D** | **3.5Dv5** | **3.5Dv3** | **3.5Dv4** |  |

Note: A *P* value no less than 0.05 is shown as numerical data. *, **, and *** denotes a *P* value less than 0.05, 0.01, and 0.005. DSC denotes Dice similarity coefficient. E&D denotes erosion and dilation. 2Da, 2Dc, 2Ds, 2.5Dv, 2.5Da, 3D, 3.5Dv5, 3.5Dv3, and 3.5Dv4 denotes 2Da U-Net, 2Dc U-Net, 2Ds U-Net, 2.5Dv U-Net, 2.5Da U-Net, 3D U-Net, 3.5Dv5 U-Net, 3.5Dv3 U-Net, and 3.5Dv4 U-Net, respectively.

Table S6. Statistical analysis of negative predictive value (NPV) among 9 U-Nets using Kruskal-Wallis test with post hoc analysis using Bonferroni correction before and after E&D.

| **NPV** | | **After E&D** | | | | | | | | |  |
| --- | --- | --- | --- | --- | --- | --- | --- | --- | --- | --- | --- |
|  |  | **2Da** | **2Dc** | **2Ds** | **2.5Dv** | **2.5Da** | **3D** | **3.5Dv5** | **3.5Dv3** | **3.5Dv4** |  |
| **Before E&D** | **2Da** |  | 1.000 | 0.997 | *** | 1.000 | * | 0.466 | *** | *** | **2Da** |
|  | **2Dc** | 1.000 |  | 0.980 | *** | 1.000 | ** | 0.290 | *** | *** | **2Dc** |
|  | **2Ds** | 1.000 | 0.981 |  | ** | 0.999 | 0.200 | 0.922 | *** | *** | **2Ds** |
|  | **2.5Dv** | *** | *** | ** |  | *** | 0.960 | 0.273 | 0.491 | 0.828 | **2.5Dv** |
|  | **2.5Da** | 1.000 | 1.000 | 1.000 | *** |  | * | 0.563 | *** | *** | **2.5Da** |
|  | **3D** | * | ** | 0.104 | 0.991 | * |  | 0.949 | * | 0.133 | **3D** |
|  | **3.5Dv5** | 0.584 | 0.278 | 0.911 | 0.280 | 0.669 | 0.868 |  | *** | ** | **3.5Dv5** |
|  | **3.5Dv3** | *** | *** | *** | 0.445 | *** | 0.054 | *** |  | 1.000 | **3.5Dv3** |
|  | **3.5Dv4** | *** | *** | *** | 0.776 | *** | 0.193 | ** | 1.000 |  | **3.5Dv4** |
|  |  | **2Da** | **2Dc** | **2Ds** | **2.5Dv** | **2.5Da** | **3D** | **3.5Dv5** | **3.5Dv3** | **3.5Dv4** |  |

Note: A *P* value no less than 0.05 is shown as numerical data. *, **, and *** denotes a *P* value less than 0.05, 0.01, and 0.005. DSC denotes Dice similarity coefficient. E&D denotes erosion and dilation. 2Da, 2Dc, 2Ds, 2.5Dv, 2.5Da, 3D, 3.5Dv5, 3.5Dv3, and 3.5Dv4 denotes 2Da U-Net, 2Dc U-Net, 2Ds U-Net, 2.5Dv U-Net, 2.5Da U-Net, 3D U-Net, 3.5Dv5 U-Net, 3.5Dv3 U-Net, and 3.5Dv4 U-Net, respectively.
